# M1 macrophage-derived exosomes and their key molecule lncRNA HOTTIP suppress head and neck squamous cell carcinoma progression by upregulating the TLR5/NF-κB pathway

**DOI:** 10.1038/s41419-022-04640-z

**Published:** 2022-02-24

**Authors:** Huaili Jiang, Lei Zhou, Na Shen, Xianhui Ning, Daquan Wu, Kanglun Jiang, Xinsheng Huang

**Affiliations:** 1 Department of Otolaryngology, Zhongshan Hospital, Fudan University, Shanghai, China; 2 Cancer Center, Zhongshan Hospital, Fudan University, Shanghai, China

**Keywords:** Cancer microenvironment, Head and neck cancer

## Abstract

Exosomes serve as a crucial mode of communication between tumor-associated macrophages (TAMs) and cancer cells. This study attempted to explore the function of M1-derived exosomes and clarify their specific mechanism in head and neck squamous cell carcinoma (HNSCC). Moreover, the functional roles of M1-derived exosomes and their key molecule long noncoding RNA (lncRNA) HOXA transcript at the distal tip (HOTTIP) in HNSCC were investigated by conducting a series of in vitro and in vivo experiments. The dual-luciferase test was utilized to clarify the binding capacities between HOTTIP/mRNA and miRNAs. Accordingly, HOTTIP was found to be upregulated in M1-derived exosomes. Meanwhile, the in vitro experiments indicated that M1 exosomes suppressed proliferation, migration and invasion but induced apoptosis of cancer cells. This function was noted to be enhanced by HOTTIP-overexpressed M1 exosomes but was weakened by HOTTIP-knockdown ones, indicating that HOTTIP serves as a key molecule in M1 exosomes. Therefore, the function of HOTTIP in cancer cells was explored, for which overexpression of HOTTIP was found to inhibit proliferation, migration and invasion but induced apoptosis of cancer cells in vitro. A mechanism study further showed that M1 exosomes and HOTTIP activated the TLR5/NF-κB signaling pathway by competitively sponging miR-19a-3p and miR-19b-3p. Furthermore, cancer cells expressing HOTTIP were noted to induce the polarization of both local M1 and M2 macrophages; however, M1 exosomes were observed to reprogram local TAMs into M1 macrophages. More importantly, both cancer cells expressing HOTTIP and M1 exosomes reeducated circulating monocytes to express the M1 phenotype. The corresponding data demonstrated that the M1 exosomal lncRNA HOTTIP suppresses HNSCC progression by upregulating the TLR5/NF-κB signaling pathway through competitively sponging miR-19a-3p and miR-19b-3p. In particular, M1 exosomes and HOTTIP induce the polarization of M1 in circulating monocytes, thus providing novel insight into HNSCC immunotherapy.

## Introduction

Head and neck cancer is the sixth most frequent malignant form of cancer globally, accounting for 5% of all malignant tumors [1]. Approximately 500,000 patients are diagnosed annually, of which 350,000 succumb to the disease [2, 3]. Head and neck cancer is a heterogeneous entity comprising tumors that occur in the oral cavity, oropharynx, nasopharynx, larynx, and hypopharynx, of which over 90% are squamous cell carcinomas [4]. At the time of diagnosis, sixty percent of patients are already in advanced stages (stage III or IV) [5, 6]. Although surgery, radiotherapy, and chemotherapy have been widely used treatments modalities, the 5-year survival rate of patients with head and neck squamous cell carcinoma (HNSCC) remains at only 50%, while the local recurrence rate can reach as high as 50% with a distant metastasis rate of 25% [7, 8]. Molecular targeted therapy has been widely used to treat a variety of cancers due to its effectiveness and safety. The epidermal growth factor receptor (EGFR) inhibitor cetuximab has been used to treat HNSCC for more than ten years. However, to date, no breakthroughs have been made in cancer treatment. The remission rate achieved with existing drugs targeting HNSCC is only 10–15% and has no clinical significance [9, 10]. Therefore, investigating valuable treatment targets to reduce the surgical, radiotherapy and chemotherapy rates in patients with HNSCC is urgently required, which may further improve their survival time and quality of life.

Inflammatory cells are a principal component of the tumor microenvironment (TME) [11]. Tumor-associated macrophages (TAMs) constitute up to 50% of solid tumors [12]. The importance of TAMs in tumor progression has been increasingly recognized. TAMs are highly plastic cells that can be classified into two polarized states: the classically activated M1 phenotype (proinflammatory and antitumor) and the alternatively activated M2 phenotype (anti-inflammatory and protumor) [13]. M1 TAMs have been suggested to be negatively correlated with colorectal carcinoma metastasis, while M2 TAMs are positively associated with liver and lymphatic metastasis and tumor differentiation grade [14]. Similar results have been reported in endometrial cancer, in which M2 triggered many aspects of tumor progression, including increasing the risks of myometrial invasion, angiogenesis, lymphovascular space invasion and lymph node metastasis [15]. Kovaleva et al. suggested that renal cell carcinoma patients with more CD11^+^ M1 and fewer CD206^+^ M2 have a better survival prognosis [16]. In esophageal cancer, M2 density is correlated with poor overall survival and vessel metastasis [17]. Lung adenocarcinoma, which has a higher density of CD204^+^ M2, showed increased levels of aggressiveness [18]. Troiano et al. also demonstrated that the M2-like marker CD163^+^ is predictive of poor prognosis in HNSCC [19]. In light of the above findings, we postulate that reversing M2 macrophages to the M1 phenotype may serve as a potential novel antitumor therapy.

Cross-talk between cancer cells and TAMs via cell-cell contact and/or soluble messengers is considered to be a principal mechanism in TAM polarization [20]. Changes in the cancer cell phenotype affect TAM polarization, and the polarization state of TAMs influences cancer cell progression (e.g., proliferation, apoptosis, migration and invasion) as well. Bladder cancer cells reeducate TAMs into M2 macrophages by secreting BMP4, a protein necessary to sustain a protumoral immune environment [21]. Therése Lindsten found that breast cancer cell proliferation can be suppressed by M1 conditioned media [22]. Recently, exosomes have been found to play an essential role in the communication between cancer cells and TAMs.

In most cases, exosomes derived from cancer cells have been shown to promote M2 TAM polarization [23–25]. Whereas, various studies have indicated that cancer-derived exosomes possess the potential to lead macrophages toward the M1 phenotype [26–28]. M1-derived exosomes suppress tumor progression through the caspase3 pathway [29]. On the contrary, exosomal miR-21 and miR-155 from M2 promote colon cancer metastasis by downregulating BRG1 [30]. However, cross-talk between HNSCC and TAMs remains unexplored.

Long noncoding RNA (lncRNA) HOXA transcript at the distal tip (HOTTIP) is located on chromosome 7 and is transcribed from the 5’ end of the HOXA locus, which can activate multiple HOXA genes. HOTTIP is related to the occurrence and development of various malignant tumors, such as liver cancer [31], pancreatic cancer [32], esophageal squamous cell carcinoma [33] and lung small cell carcinoma [34] and is considered an essential oncogene. However, A recent study has found that HOTTIP also possesses antitumor properties, which shows that HOTTIP inhibits glioma proliferation by promoting apoptosis by downregulating the BRE gene [35]. These findings illustrate that HOTTIP acts in a bidirectional manner regarding the progression of different cancers. Similarly, the bidirectional functions of exosomal HOTTIP have also been reported. For example, Zhao et al. suggest that the increased serum exosomal HOTTIP levels are significantly correlated with gastric cancer patients’ poor overall survival of gastric cancer patients [36]. However, Oehme et al. demonstrate a significant correlation between low levels of serum exosomal HOTTIP and poor overall survival of colorectal cancer patients [37]. Therefore, clarifying the underlying role and mechanism of HOTTIP in HNSCC is necessary and can provide meaningful insight into its nature.

Due to the great potential of M1 exosomes in tumor treatment and the gap between M1 exosomes or HOTTIP and HNSCC research, this study attempts to explore the function and clarify the mechanism of M1 exosomal HOTTIP in HNSCC. Furthermore, this study investigates their influences on TAM polarization in the TME. Accordingly, the present study’s findings may provide novel insight into immunotherapy for HNSCC.

## Results

### The correlation between TAMs and HNSCC prognosis and the establishment of HOTTIP-overexpressed and -knockdown exosomes in M1

**Fig. 1:**
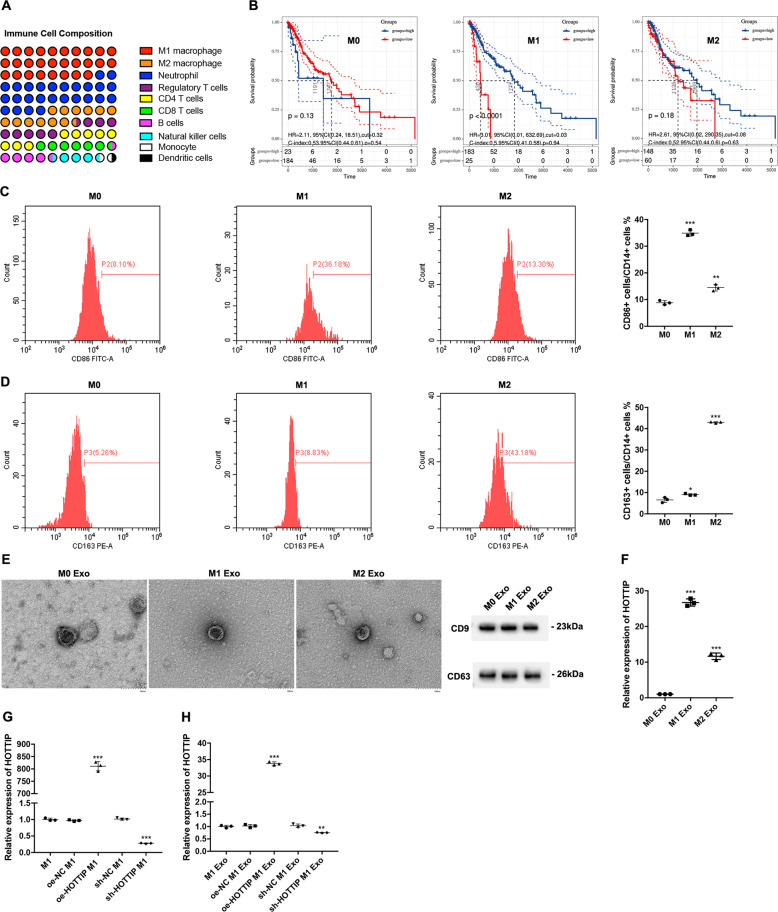
The correlation between TAMs and HNSCC prognosis and the establishment of HOTTIP overexpression and knockdown exosomes in M1. **A** The infiltration levels of immune cells in HNSCC. **B** Prediction of overall survival according to high- and low-infiltration levels of M0, M1 and M2 macrophages. THP-1-derived M0, M1 and M2 macrophages were confirmed by flow cytometry using (**C**) CD14 plus CD86 and (**D**) CD14 plus CD163 (*n* = 3). **E** The exosomes were extracted from three types of macrophages and confirmed by electronic microscopy and Western blot analysis using CD9 and CD63. **F** RT-qPCR was conducted to test the expression of HOTTIP in M0, M1 and M2 macrophages (*n* = 3). RT-qPCR was conducted to test the expression of HOTTIP in wild type M1 macrophages, HOTTIP-overexpressed and HOTTIP-knockdown M1 macrophages (**G**) and their exosomes (**H**) (*n* = 3). Data are presented as mean ± SD. Results were analyzed by One-way ANOVA with a post hoc *t*-test. Significance: *P < 0.05, **P < 0.01, ***P < 0.001.

Bioinformatics analysis demonstrated that HNSCC was infiltrated by several immune cells, of which M1 macrophages accounted for the most (28.2%), followed by neutrophils (25.6%) and M2 macrophages (12.8%) (Fig. 1A). The M0 (*p* = 0.13, HR = 2.11, 95% CI: 0.24–18.51) and M2 (*p* = 0.18, HR = 2.61, 95% CI: 0.02–290.35) infiltration levels were not noted to influence the overall survival of HNSCC patients; however, higher M1 infiltration levels (*p* < 0.0001, HR = 3.01, 95% CI: 0.01–632.69) were associated with a better survival prognosis (Fig. 1B). The THP-1 cells were induced into M0 (CD14^high^CD86^low^CD163^low^), M1(CD14^high^CD86^high^CD163^low^) and M2 (CD14^high^CD86^low^CD163^high^) macrophages, respectively, which were then confirmed by flow cytometry (Fig. 1C, D). The exosomes derived from M0, M1 and M2 macrophages were extracted and confirmed by electronic microscopy and Western blotting utilizing CD9 and CD63 (Fig. 1E). Furthermore, HOTTIP expression in exosomes was analyzed, which showed it was significantly higher in M1 exosomes than either in M0 or M2 exosomes (Fig. 1F). In light of the above results, HOTTIP-overexpressed and -knockdown M1 macrophages (Fig. 1G) were established, after which their exosomes were extracted. HOTTIP expression in exosomes derived from six types of macrophages was confirmed (Fig. 1H).

### M1 exosomes inhibit HNSCC progression in vitro with HOTTIP being a key molecule

**Fig. 2:**
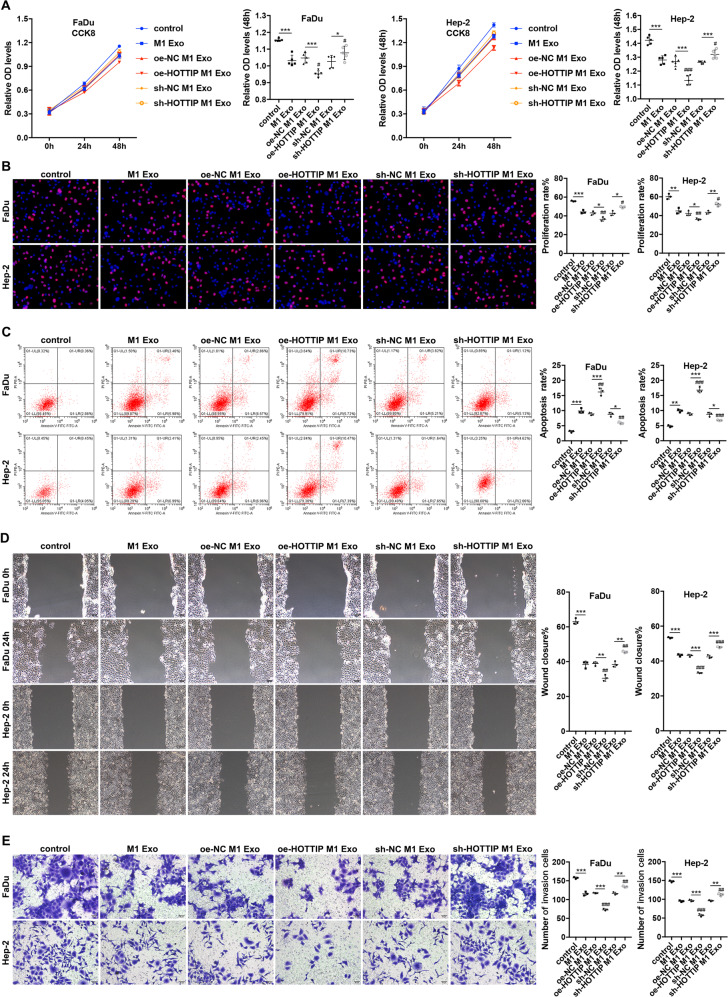
M1-derived exosomes inhibit HNSCC progression in vitro with HOTTIP being a key molecule. **A** CCK8 assay evaluated the cell viability of FaDu and Hep-2 cells at 24 h and 48 h after being treated by different types of M1 exosomes (*n* = 5). HOTTIP-overexpressed M1 exosomes inhibited cell viability while HOTTIP-knockdown ones promoted it compared to wild-type M1 exosomes. **B** EdU assay evaluated the proliferation capacity of FaDu and Hep-2 cells following treatment by different types of M1 exosomes (*n* = 3). HOTTIP-overexpressed M1 exosomes suppressed cell proliferation capacity while sh-HOTTIP ones promoted it compared to wild-type M1 exosomes. **C** Apoptosis assay. FaDu and Hep-2 cells were incubated with FITC labeled Annexin V antibody and were then stained by PI following treatment by different types of M1 exosomes (*n* = 3). The apoptosis rates were determined by flow cytometry. HOTTIP-overexpressed M1 exosomes increased the apoptosis rate while HOTTIP-knockdown ones decreased it compared to wild-type M1 exosomes. Scratch test (**D**) and Transwell assay (**E**) examined the migration and invasion ability of FaDu and Hep-2 cells after treatment by different types of M1 exosomes (*n* = 3). HOTTIP-overexpressed M1 exosomes inhibited cell migration and invasion ability while HOTTIP-knockdown ones promoted it compared to wild-type M1 exosomes. Data are presented as mean ± SD. Results were analyzed by *t*-tests and One-way ANOVA with a post hoc test. * compared to control or their NC, # compared to M1 exosomes. Significance: */#*P* < 0.05, **/##*P* < 0.01, ***/###*P* < 0.001.

To explore the function of M1 exosomes, various in vitro experiments were conducted. We used THP-1-differentiated M1 macrophages to establish HOTTIP-overexpressed, HOTTIP-knockdown M1 macrophages and their negative control ones. Further, we extracted their exosomes separately. Accordingly, the CCK-8 and EdU assay indicated that M1 exosomes suppressed cell proliferation of both FaDu and Hep-2 cells and this function was noted to be enhanced by HOTTIP-overexpressed M1 exosomes but was weakened by HOTTIP-knockdown ones (Fig. 2A, B). Flow cytometry analysis showed that the highest apoptosis rate was present in the group of HOTTIP-overexpressed M1 exosomes, followed by that of wild type and HOTTIP-knockdown ones (Fig. 2C). Scratch tests illustrated that, after 24 h, M1 exosomes suppressed the wound closure rate compared to that of the control group. HOTTIP-overexpressed M1 exosomes significantly decreased the wound closure rate; however, HOTTIP-knockdown ones increased the rate compared to M1 exosomes or their empty vector group (Fig. 2D). Transwell assays revealed that, after 24 h, M1 exosomes inhibited the invasion ability of cancer cells compared to that of the control group. HOTTIP-overexpression M1 exosomes further restrained invasion ability; however, HOTTIP-knockdown ones were shown to promote it compared to M1 exosomes or their empty vector group (Fig. 2E). The data suggested that M1 exosomes could inhibit HNSCC progression by suppressing proliferation, migration and invasion and inducing apoptosis via HOTTIP. Therefore, HOTTIP was a key molecule in M1 exosomes.

### HOTTIP suppresses HNSCC progression in vitro

**Fig. 3:**
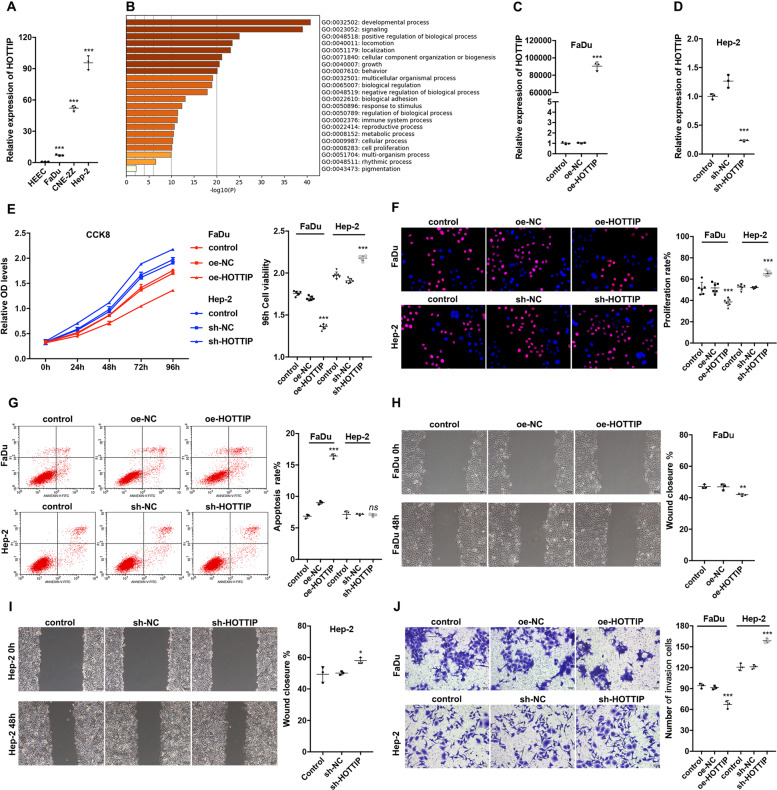
HOTTIP suppresses HNSCC progression in vitro. **A** RT-qPCR detected the relative expression of HOTTIP in three HNSCC cell lines compared to those in HEEC cells (*n* = 3). **B** Top 22 Gene Ontology processes following analysis of 3000 HOTTIP-associated genes. Efficiency of HOTTIP expression in HOTTIP-overexpressed FaDu cells (**C**) and HOTTIP-knockdown Hep-2 cells (**D**) were confirmed using RT-qPCR (*n* = 3). **E** CCK8 assay detected the cell viability after HOTTIP overexpression or knockdown. Overexpression of HOTTIP inhibited the cell viability of FaDu cells but knockdown of HOTTIP promoted the cell viability of Hep-2 cells at 24, 48, 72 and 96 h (*n* = 6). **F** EdU assay evaluated the proliferation capacity of HOTTIP-overexpressed FaDu and HOTTIP-knockdown Hep-2 cells. Overexpression of HOTTIP inhibited the cell proliferation capacity of FaDu cells but knockdown of HOTTIP promoted that of Hep-2 cells (*n* = 6). **G** Apoptosis assay. HOTTIP-overexpressed FaDu and HOTTIP-knockdown Hep-2 cells were incubated with FITC labeled Annexin V antibody and were then stained by PI. The apoptosis rates were determined by flow cytometry. Overexpression of HOTTIP induced apoptosis but knockdown of HOTTIP did not influence apoptosis (*n* = 3). Scratch test (**H, I**) and Transwell assay (**J**) examined the migration and invasion ability of HOTTIP-overexpressed FaDu and HOTTIP-knockdown Hep-2 cells. Overexpression of HOTTIP inhibited the cell migration and invasion ability of FaDu cells but knockdown of HOTTIP promoted that of Hep-2 cells (*n* = 3). Data are presented as mean ± SD. Results were analyzed by One-way ANOVA with a post hoc test. Significance: *P < 0.05, **P < 0.01, ***P < 0.001.

After being released by macrophages, cancer cells could take up exosomes, with HOTTIP being the key molecule functioning in cancer cells. Therefore, the function of HOTTIP in cancer cells was further explored. First, HOTTIP expression was evaluated in three HNSCC cell lines. It was most upregulated in Hep-2 cells, followed by CNE-2Z and FaDu cells, compared to HECC cells (Fig. 3A). Next, a Gene Ontology (GO) analysis of 3000 HOTTIP-associated genes was conducted, in which HOTTIP was noted to be responsible for both the positive and negative regulation of biological processes and immune system processes (Fig. 3B). To clarify the precise function of HOTTIP in vitro, HOTTIP-overexpressed FaDu cells and HOTTIP-knockdown Hep-2 cells were established, respectively (Fig. 3C, D). Here, the CCK-8 and EdU assays indicated that HOTTIP overexpression inhibited cancer cell proliferation, though HOTTIP knockdown induced proliferation (Fig. 3E, F). Flow cytometry analysis showed that HOTTIP overexpression triggered the apoptosis of cancer cells; however, its knockdown had no influence (Fig. 3G). Moreover, scratch tests found that HOTTIP overexpression inhibited, but HOTTIP knockdown promoted migration ability compared to their empty vector group after 48 h (Fig. 3H, I). Transwell assays illustrated that HOTTIP overexpression inhibited; however, HOTTIP knockdown promoted invasion capacity compared to their empty vector group (Fig. 3J). The data suggested that HOTTIP suppressed HNSCC progression by inhibiting proliferation, migration and invasion while partially inducing apoptosis.

### M1 exosomes and HOTTIP suppressed HNSCC progression in vivo

**Fig. 4:**
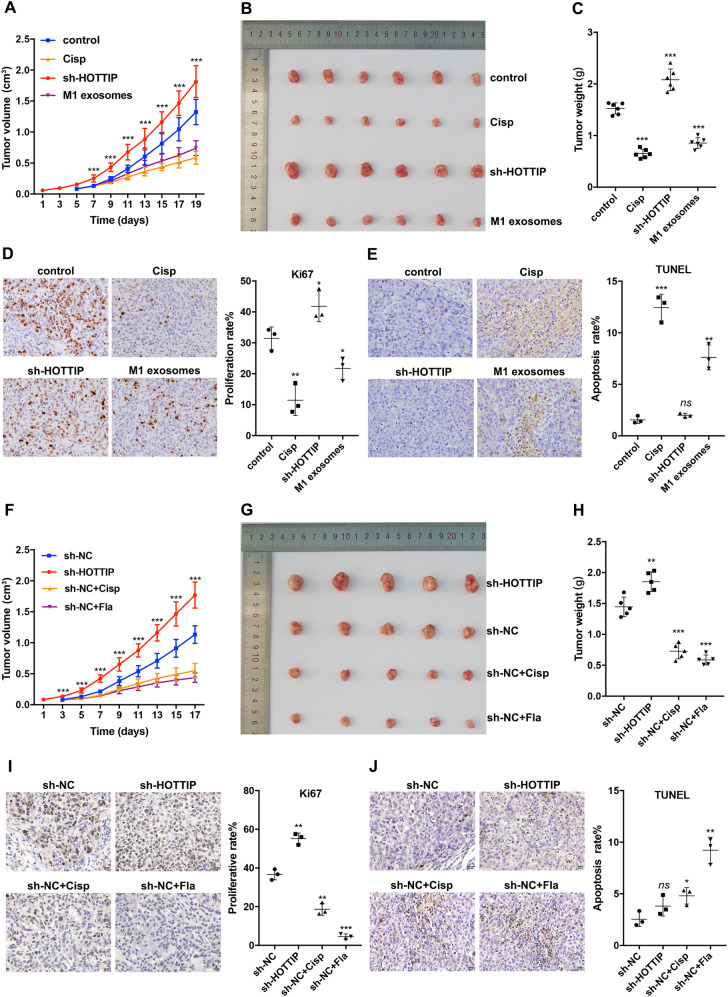
M1 exosomes and HOTTIP inhibit HNSCC progression in vivo. **A** The Balb/c nude mice were subcutaneously injected with HOTTIP-knockdown Hep-2 cells or wild type Hep-2 cells, which follow-up accepted treatment of normal saline, cisplatin or M1 exosomes (*n* = 6 per group) every other day for 14 days. Tumors were removed following 14-day treatment. Tumor volume (**B**) and tumor weight (**C**) were analyzed in each group. **D** Immunohistochemistry (IHC) detected proliferation capacity using Ki67 staining, showing that M1 exosomes and cisplatin inhibited proliferation capacity while knockdown of HOTTIP promoted it (*n* = 3). **E** IHC detected the apoptosis rate using TUNEL staining, which indicated that M1 exosomes and cisplatin increased the apoptosis rate while knockdown of HOTTIP did not influence it (*n* = 3). **F** The Balb/c nude mice were subcutaneously injected with HOTTIP-knockdown Hep-2 cells or sh-NC Hep-2 cells, which follow-up accepted treatment of normal saline, cisplatin or flagellin (*n* = 5 per group) every other day for 14 days. Tumors were removed following 14-day treatment. Tumor volume (**G**) and tumor weight (**H**) were analyzed in each group. **I** IHC detected proliferation capacity using Ki67 staining, showing that knockdown of HOTTIP facilitated tumor proliferation while cisplatin and flagellin suppressed it (*n* = 3). **J** IHC detected the apoptosis rate using TUNEL staining, which indicated that cisplatin and flagellin increased the apoptosis rate while knockdown of HOTTIP did not influence it (*n* = 3). Data are presented as mean ± SD. Results were analyzed by One-way ANOVA with a post hoc test. Significance: *P < 0.05, **P < 0.01, ***P < 0.001.

Based on the in vitro results, an animal model was established in Balb/c nude mice to explore the function of exosomes derived from THP-1-differentiated M1 macrophages. Cisplatin and Flagellin (a specific agonist of TLR5) were utilized as a positive control of tumor killing. Accordingly, the tumors induced by M1 exosomes exhibited a lower growth rate (Fig. 4A), smaller volumes (Fig. 4B) and lighter tumor weights (Fig. 4C) than those triggered by NS (control). IHC staining revealed significantly fewer Ki67^+^ cells in M1 exosome-induced xenografts than in the control group (Fig. 4D). The apoptosis rates were also significantly increased in xenografts accepting M1 exosome induction (Fig. 4E). These findings suggested that M1 exosomes inhibited cancer cell proliferation but triggered apoptosis in vivo.

In addition, another animal model was established using stable HOTTIP-knockdown Hep-2 cells. The tumors stemming from HOTTIP-knockdown cells demonstrated a higher growth rate (Fig. 4F), more significant volumes (Fig. 4G) and heavier tumor weights (Fig. 4H). Moreover, those accepting cisplatin or flagellin induction had smaller volumes (Fig. 4G) and lighter tumor weights (Fig. 4H). IHC staining revealed that significantly more Ki67^+^ cells were detected in HOTTIP-knockdown xenografts than in the other three groups (Fig. 4I), which suggested that HOTTIP inhibited cancer cell proliferation in vivo. The apoptosis rates were shown to be increased in xenografts treated by cisplatin or flagellin; however, HOTTIP knockdown was not noted to affect apoptosis (Fig. 4J). The above data suggested that HOTTIP inhibited cancer cell proliferation in vivo. Taken together with those coming from cells lines, HOTTIP serves as a tumor suppressor in HNSCC.

### HOTTIP and M1 exosomes activated the TLR5/NF-κB signaling pathway through competitive binding of miR-19a-3p and miR-19b-3p

**Fig. 5:**
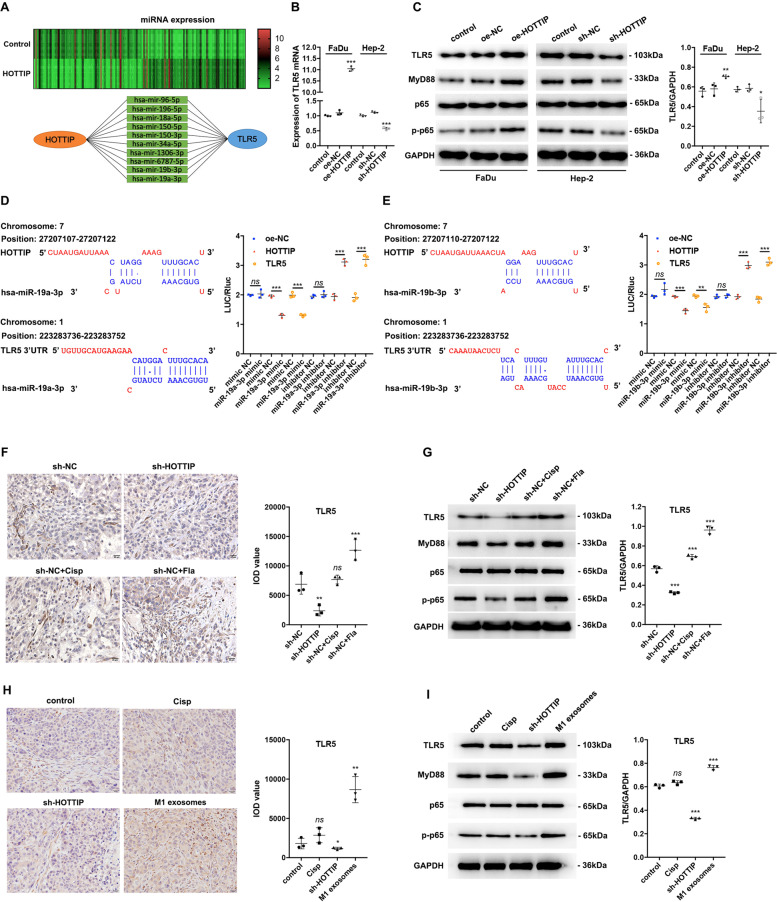
HOTTIP and M1 exosomes upregulate the TLR5/NF-κB signaling pathway by competitively sponging miR-19a-3p and miR-19b-3p. **A** High-level Agilent miRNA chip was established using HOTTIP-overexpressed FaDu-derived exosomes. The expression of 5000 miRNAs was tested, of which 257 miRNAs were significantly downregulated (upper panel). Ten potential ceRNA networks were predicted by the miRWalk 2.0 database (lower panel). **B** RT-qPCR detected the relative expression of TLR5 mRNA. Overexpression of HOTTIP upregulated TLR5 mRNA expression while knockdown of HOTTIP downregulated it (*n* = 3). **C** Western blot assay of a key protein (TLR5, MyD88, p65 and p-p65) in TLR5/NF-κB signaling pathway. Overexpression of HOTTIP upregulated TLR5 protein expression while knockdown of HOTTIP downregulated it (*n* = 3). The predicted binding site between HOTTIP, miR‐19a-3p (**D**)/miR‐19b-3p (**E**) and TLR5 mRNA. HEK293T cells were transfected with miR-19a/b-3p mimic or inhibitor. Relative luciferase activity (LUC/Rluc) was detected 48 h post interaction using dual luciferase assay. miR-19a/b-3p mimic decreased LUC/Rluc while miR-19a/b-3p inhibitor increased it (*n* = 3). IHC detected TLR5 protein expression, which suggested that knockdown of HOTTIP (**F**) downregulated while M1 exosomes (**H**) upregulated TLR5 expression (*n* = 3). Western blot assay of a key protein in TLR5/NF-κB signaling pathway. Knockdown of HOTTIP (**G**) downregulated while M1 exosomes (**I**) upregulated TLR5 expression (*n* = 3). Data are presented as mean ± SD. Results were analyzed by One-way ANOVA with a post hoc test. Significance: *P < 0.05, **P < 0.01, ***P < 0.001.

To clarify the mechanism by which HOTTIP suppressed HNSCC progression, a high-level miRNA chip utilizing exosomes extracted from HOTTIP-overexpressed FaDu cells was established. The expression of 5000 miRNAs was tested, of which 257 miRNAs were significantly downregulated (Fig. 5A upper panel). Next, miRWalk 2.0 database was used to predict their potential target genes. Finally, ten potential ceRNA networks were selected for further verification utilizing a dual-luciferase reporter system (Fig. 5A lower panel). The expression of TLR5 mRNA was found to be increased in HOTTIP-overexpressed FaDu cells but was decreased in HOTTIP-knockdown Hep-2 cells (Fig. 5B). Then, the protein levels of the TLR5/NF-κB signaling pathway were further detected, illustrating that TLR5, MyD88, p-p65 and p-p65/p65 were increased in HOTTIP-overexpressed FaDu cells but were decreased in HOTTIP-knockdown Hep-2 cells, which suggested that HOTTIP activated the TLR5/NF-κB signaling pathway (Fig. 5C and Supplementary Fig. 1A). In contrast to NC, the relative luciferase activities of the miR-19a-3p mimic (Fig. 5D) and miR-19b-3p mimic (Fig. 5E) in either HOTTIP or TLR5 wild-type cells were found to be decreased. Simultaneously, the relative luciferase activities of the miR-19a-3p (Fig. 5D) inhibitor and miR-19b-3p inhibitor (Fig. 5E) in either HOTTIP or TLR5 wild-type cells were high. However, the relative luciferase activities of the miR-96-5p, miR-196-5p, miR-18a-5p, miR-150-5p, miR-150-3p, miR-34a-5p, miR-1306-3p and miR-6787-5p mimic did not exhibit the expected change in trend (Supplementary Fig. 2A-H).

In the nude mice animal model, IHC staining detected a decrease in TLR5 protein expression (Fig. 5F). Similarly, Western blot analysis detected a decrease in TLR5, MyD88, and p-p65 protein (Fig. 5G and Supplementary Fig. 1B) in xenografts stemming from HOTTIP-knockdown cells compared to those from sh-NC or induced by flagellin, which is a specific agonist of the TLR5 receptor. Similar results were noted in another nude mice animal model, where TLR5 protein levels were found to be increased following treatment with M1 exosomes compared to that of the control group (Fig. 5H). Western blot revealed elevations in TLR5, MyD88, and p-p65 protein expression (Fig. 5I and Supplementary Fig. 1C) in xenografts treated with M1 exosomes compared to the control group. Meanwhile, cisplatin did not influence the expression of TLR5/NF-κB signaling pathway. Therefore, these data demonstrated that HOTTIP and M1 exosomes activated the TLR5/NF-κB signaling pathway by sponging miR-19a-3p and miR-19b-3p.

### HOTTIP triggered the secretion of proinflammatory cytokines

The expression of inflammatory cytokines was tested at 0 h, 48 h and 72 h via ELISA, which showed the concentrations of TNF-α, IL-6 and IL-1β were increased in HOTTIP-overexpressed FaDu cells while that of IL-10 decreased (Supplementary Fig. 3A). In HOTTIP-knockdown Hep-2 cells, the opposite was observed for TNF-α, IL-6 and IL-10 (Supplementary Fig. 3B), but no significant change of IL-4 was detected in either cell line. The data suggested that cancer cells expressing HOTTIP rendered the TME proinflammatory.

### Cancer cells expressing HOTTIP and M1 exosomes regulated the polarization of TAMs

**Fig. 6:**
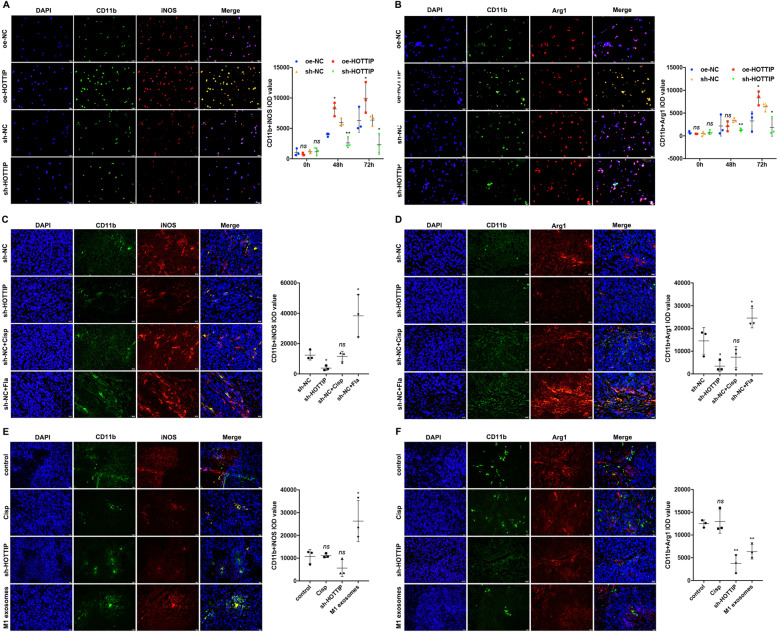
Cancer cells expressing HOTTIP promote the polarization of local TAMs towards both M1 and M2, but M1 exosomes reeducate TAMs to M1. Immunofluorescence (IF) staining assay determined CD11b^+^iNOS^+^ M1 (**A**) or CD11b^+^Arg1^+^ M2 (**B**) phenotype 48 and 72 h post co-culture of cancer cells (upper chamber) and THP-1-derived M0 macrophages (lower chamber). HOTTIP-overexpressed FaDu cells induced polarization while HOTTIP-knockdown Hep-2 cells inhibited polarization of M0 macrophages into both M1 and M2 phenotypes (*n* = 3). IF staining detected M1 (**C**) or M2 (**D**) phenotype in tumors derived from HOTTIP-knockdown Hep-2 cells. HOTTIP-knockdown Hep-2 cells suppressed the polarization of M0 macrophages into both M1 and M2 phenotypes (*n* = 3). IF staining detected M1 (**E**) or M2 (**F)** phenotype in tumors derived from Hep-2 cells treated by M1 exosomes. M1 exosomes induced the polarization of M0 macrophages into the M1 phenotype but inhibited that of M0 ones into the M2 phenotype (*n* = 3). Data are presented as mean ± SD. Results were analyzed by One-way ANOVA with a post hoc test. Significance: *P < 0.05, **P < 0.01, ***P < 0.001.

According to the inflammatory cytokine results, the polarization of TAMs both in vitro and in vivo was further explored. We established a Transwell co-culture system in which cancer cells were in the upper chamber and THP-1-derived M0 macrophages were in the lower chamber, which revealed that HOTTIP-overexpressed FaDu cells promoted, while HOTTIP-knockdown Hep-2 cells decreased, the number of CD11b^+^iNOS^+^ M1 macrophages after 72 h (Fig. 6A). However, the amount of M2 exhibited the same change in trend: HOTTIP-overexpressed FaDu cells were promoted, while HOTTIP-knockdown Hep-2 cells decreased, the number of CD11b^+^Arg1^+^ M2 macrophages following 72 h (Fig. 6B). Similarly, the number of CD11b^+^iNOS^+^ M1 and CD11b^+^Arg1^+^ M2 macrophages also decreased in HOTTIP-knockdown xenografts, which was in line with the in vitro results (Fig. 6C, D). Interestingly, in another nude mice animal model, M1 exosomes reeducated TAMs into CD11b^+^iNOS^+^ M1 macrophages (Fig. 6E), but they inhibited the polarization of CD11b^+^Arg1^+^ M2 ones (Fig. 6F). These data suggested that cancer cells expressing HOTTIP polarized TAMs into macrophages mixed phenotype of M1 and M2, whereas M1 exosomes reprogrammed TAMs into the M1 phenotype.

### Cancer cells expressing HOTTIP and M1 exosomes regulated the polarization of circulating monocytes

**Fig. 7:**
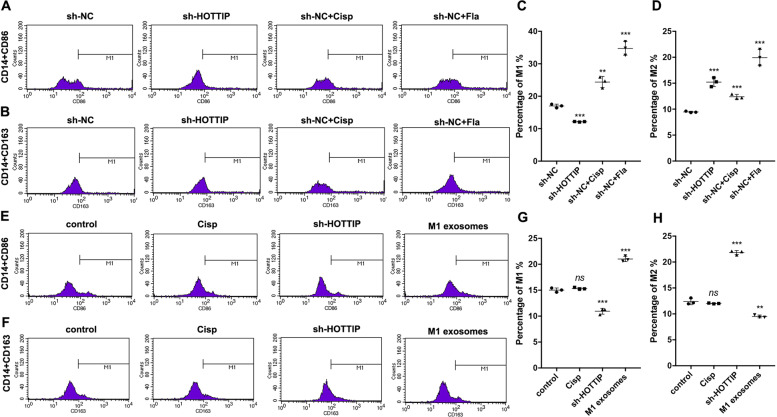
Cancer cells expressing HOTTIP and M1 exosomes reprogram the circulating monocytes into M1 phenotype. **A–D** Flow cytometry assay detected CD14^+^CD86^+^ M1 and CD14^+^CD163^+^ M2 phenotype in circulating blood of nude mice bearing with HOTTIP-knockdown Hep-2 cells (*n* = 3). HOTTIP-knockdown Hep-2 cells suppressed the polarization of M1 monocytes (**A**, **C**) but induced the polarization of M2 (**B**, **D**). **E**–**H** Flow cytometry assay detected M1 and M2 phenotypes in the blood of tumor-bearing nude mice treated by M1 exosomes (*n* = 3). M1 exosomes induced the polarization of M1 monocytes (**E**, **G**) but inhibited the polarization of M2 (**F**, **H**). Data are presented as mean ± SD. Results were analyzed by One-way ANOVA with a post hoc test. Significance: *ns* not significant, **P < 0.01, ***P < 0.001.

The polarization of monocytes in circulating blood from two animal models was also analyzed. Circulating CD14^+^CD86^+^ M1 monocytes decreased in nude mice bearing HOTTIP-knockdown xenografts compared to those bearing sh-NC xenografts (Fig. 7A, C), while CD14^+^CD163^+^ M2 monocytes increased (Fig. 7B, D). These results were consistent with another nude mice animal model, in which the number of circulating CD14^+^CD86^+^ M1 monocytes increased in mice accepting M1 exosome induction compared to those accepting NS treatment (Fig. 7E, G), while CD14^+^CD163^+^ M2 monocytes decreased (Fig. 7F, H). The data suggested that cancer cells expressing HOTTIP and M1 exosomes reeducated circulating monocytes into the M1 phenotype.

## Discussion

In the present study, M1-derived exosomes and their key molecule lncRNA HOTTIP were found to suppress the progression of HNSCC in two ways. Specifically, M1 exosomes and HOTTIP inhibited tumor cells’ progression while activating the innate immune system. Most importantly, the two paths promoted each other and formed a closed positive feedback loop.

Solid tumors are composed of tumor cells and numerous nontumor cells, of which most are immune cells. TAMs are predominantly immune cells that coordinate various factors in the TME [38]. Accordingly, in HNSCC tissues, macrophages, including M1 and M2 macrophages, were noted to account for an average of 38.4% in all immune cells, indicating that macrophages may play an essential role in HNSCC progression. Hence, the interaction between tumor cells and macrophages in a cell-to-cell or soluble manner may significantly affect tumor progression (e.g., proliferation, apoptosis, metastasis and invasion). Exosomes serve as an essential medium of tumor and immune cell communication as they can transport proteins and RNAs and glycoconjugates, lipids, and DNAs [39]. However, no studies have explored the interaction between HNSCC cells and TAMs. Based on the above theory, exosomes were isolated from antitumor M1 macrophages, in which M1 exosomes were confirmed to inhibit HNSCC progression. Furthermore, M1 exosomes were confirmed to render TME proinflammatory properties and reeducate TAMs into M1 macrophages, constituting a positive feedback regulation pathway. Most importantly, this study determined that lncRNA HOTTIP served as a crucial point in M1 exosomes, for which its mechanism was further clarified.

Most studies have reported HOTTIP to be an oncogene. However, in the present study, we suggested HOTTIP act as a tumor suppressor in HNSCC, playing a similar role as it does in glioma. In addition to its inhibitory effect in cancers, HOTTIP was also found to decrease the proliferation and cell migration in Hirschsprung disease [40]. This finding may be attributed to the fact that lncRNAs show tissue-specific expression patterns and play different roles in different diseases, including cancers [41]. For instance, lncRNA TUG1 acts as an oncogene in hepatocellular carcinoma via promotion of cell growth [42]; however, TUG1 acts as a tumor suppressor in human glioma by inducing cell apoptosis [43]. Similarly, lncRNA H19 can both promote and suppress tumors [44, 45].

Both HOTTIP overexpression and M1 exosomes promoted cancer cell apoptosis significantly. Specifically, HOTTIP knockdown did not further reduce the apoptosis rate of Hep-2 cells since the control and sh-NC cells were already in a state of a meager apoptosis rate (~7.13%). Therefore, HOTTIP is believed to suppress HNSCC progression by promoting cell apoptosis. Mechanistically, the present study suggests that HOTTIP upregulates the TLR5/NF-κB signaling pathway by competitively binding to miR-19a-3p and miR-19b-3p, thereby suppressing HNSCC progression.

Inflammatory cytokines are secreted by immune cells and play a crucial role in TME and TAMs’ polarization. M1 macrophages release proinflammatory cytokines such as IL-1β, IL-6, and TNF-α to activate innate immunity and kill tumor cells [46]. Moreover, M2 ones produce the anti-inflammatory cytokine IL-10 to promote tumor development [47]. Interestingly, HOTTIP upregulated the production of proinflammatory cytokines (IL-1β, IL-6, TNF-α), though it downregulated anti-inflammatory cytokines (IL-10). Once peripheral blood monocytes are recruited to the tumor, they rapidly differentiate into the M2 macrophages via induction of IL-4 and IL-10 derived from tumor cells [48, 49], Th2-polarized CD4^+^ cells [50] and regulatory T cells [51]. Most importantly, the release of proinflammatory cytokines during the early stage of oncogenesis favors the recruitment and polarization of M1 ones [52]. These results suggested that HOTTIP can activate the internal immune function of tumor cells and kill themselves.

Although M1 and M2 macrophages confer opposing effects on tumor progression, the findings of this study revealed that cancer cells expressing HOTTIP increased the amounts of both M1 and M2 macrophages in vitro and in the nude mice animal model. This may be due to three potential explanations: (i) M1 and M2 have different origins. TAMs were previously thought to be solely recruited from bone marrow; however, TAMs can also be differentiated from tissue-resident macrophages, including Kupffer cells, peritoneal macrophages, epidermal Langerhans cells and lung alveolar macrophages [53]. Regrettably, the resident macrophages in HNSCC have yet to be fully understood. Nevertheless, recruited and resident macrophages usually coexist, though recruited macrophages constitute the majority. (ii) M1 and M2 coexist in cancers. This was also reported in murine fibrosarcoma [54], sarcoma [55] and cutaneous squamous cell carcinoma [56], which may depend on the balance between eosinophil-delivered IL-13 and overall amounts of TLRs, TNFR and IL-1R signaling [57]. (iii) Another important reason is that M1 and M2 markers are expressed in the same cells. For example, Müller et al. found that in gliomas, blood-derived TAMs frequently coexpress M1 and M2 genes in individual cells via large-scale transcriptome analysis [58]. However, the specific function of mixed phenotype TAMs remains unclear and requires further elucidation.

Additionally, the polarization of circulating monocytes was explored, demonstrating that cancer cells expressing HOTTIP and M1 exosomes reeducate them into M1 in mice circulating blood. Few studies exist on circulating monocytes; however, they all possess unique advantages in clinical application. For example, Guo et al. suggested that the different polarization states of circulating monocytes may serve as potential serum biomarkers to diagnose and monitor glioma [59]. Similar results were reported in colorectal cancer, in which circulating monocytes educated by tumor cells can be a robust biomarker for diagnosis and follow-up [60].

Notably, although studies on TAM polarization have strictly divided macrophages into two extremes (M1 and M2), macrophages are very plastic and sensitive to changes within TME Macrophages may actually exist as a continuum of phenotype and functional states in vivo, where the two ends of the continuum are defined as M1 and M2 [61]. Therefore, the tense distinction between M1 and M2 does not fully represent the continuum of functional states of macrophages [62].

The Balb/c nude mice, which were immunodeficient, were used to establish the HNSCC model. For our study focused on the tumor microenvironment, it would be better to build an animal model in immunocompetent animals. The ideal choice was to establish a spontaneous model in humanized mice which was accepted for injecting CD34^+^ or engrafting with human hematopoietic stem cells or human peripheral blood mononuclear cells in immunodeficient mice. This kind of animal model has a functional human immune system, and displays T-cell dependent inflammatory responses, with no donor cell immune reactivity toward host model [63]. However, this model has the longest research span, over 12 months and the application of humanized CD34^+^ mice in HNSCC has not been reported till now. Another choice was to establish syngeneically transplanted models by giving wild type C57Bl/6 mice 4NQO in drinking water for 16 weeks and then regular water until week 22. Cells were isolated from the lesions, cultured, and then orthotopically implanted into the tongue of wild-type C57Bl/6 mice [64]. However, this method has no reports of successful establishment of laryngeal or hypopharyngeal squamous cell carcinoma. Meanwhile, these kinds of animal models involve many animals and much time. Therefore, due to these limitations, the spontaneous HNSCC model can hardly be employed widely [65]. Further research is warranted to develop better animal models.

## Conclusions

In summary, M1 exosomes and their key molecules HOTTIP suppress HNSCC progression through upregulation of the TLR5/NF-κB signaling pathway by competitively sponging miR-19a-3p and miR-19b-3p. Significantly, M1 exosomes and HOTTIP polarize circulating monocytes into the antitumor M1 phenotype, which may provide novel insight into HNSCC immunotherapy.

## Materials and methods

### In silico analysis

Data on immune cell infiltration ratio and score (Cibersort LM22) were downloaded from The Cancer Immunome Database (TCIA) [66]. Clinical information and gene expression data for HNSCC were acquired from The Cancer Genome Atlas (TCGA). The high/low macrophages infiltration levels were determined with the optimal cutoff value calculated by the maxstat package of R software. Kaplan–Meier plots were generated using Sangerbox tools (http://www.sangerbox.com/tool). Gene Ontology (GO) analysis was performed for 3000 lncRNA HOTTIP-associated genes via Metascape [67]. The miRWalk2.0 database was then used to predict the targeted genes of miRNAs [68].

### Cell lines

The head and neck squamous cell carcinoma cell lines FaDu, CNE-2Z and Hep-2; the human esophageal epithelial cell line HEEC; human myeloid leukemia mononuclear cells THP-1; and HEK293T cells were purchased from ATCC. The cells were cultured according to the instructions provided by the ATCC. These cells were characterized by Genewiz Inc. (China) utilizing short tandem repeat (STR) markers and tested for free of mycoplasma.

### Plasmid construction and transfection

HOTTIP (oe-HOTTIP) plasmid and controls (oe-NC) were synthesized utilizing pcDNA3.1.

Next, the plasmids were transfected into THP-1-differentiated M1 macrophages or FaDu cells using Lipofectamine 3000 (L3000015, Invitrogen, USA) according to the manufacturer’s manual. One shRNA sequence targeting HOTTIP (sh-HOTTIP GGATTTGTCCTGACCAATGTA) was built and a universal sequence for HOTTIP (NC, nonsense control: TTCTCCGAACGTGTCACGT) was designed. It was incorporated into pLenR-GPH lentivirus vectors to introduce the shRNA into cells. The M1 macrophages or Hep-2 cells were infected by 8 µl lentiviral virus. The Hep-2 cell lines with stable HOTTIP interference expression and nonsense control were screened out using puromycin (5 μg/ml).

### Macrophage culture and polarization

M0 macrophages were obtained by treating THP-1 cells with 100 ng/ml phorbol 12-myristate 13-acetate (PMA, HY-18739, MedChemExpress, USA) for 48 h. Then, M0 macrophages were induced with 100 ng/ml bacterial lipopolysaccharide (LPS, HY-D1056, MedChemExpress, USA) + 2.5 ng/ml IFN-γ (C014, novoprotein, China) for 48 h to acquire the M1 phenotype. Meanwhile, M0 macrophages were induced with 10 ng/mL IL-4 (CX03, novoprotein, China) + 10 ng/mL IL-13 (CC89, novoprotein, China) for 48 h to acquire the M2 phenotype. Polarized status was confirmed utilizing flow cytometry.

### Exosome preparation and identification

M0 and M1 macrophages were then cultured in RPMI-1640 medium (SH30809.01B, Hyclone, USA) with 10% FBS + 1% P/S in a culture box at 37 °C with 5% CO_2_. After 2 days, the cell supernatant was collected and centrifuged to remove cell debris. Exosomes were isolated utilizing Total Exosome Isolation Reagent (4478359, Thermo, USA) and were confirmed to have a saucer or cup-shaped structure with a size of 30 to 150 nm by using an electron microscope [69]. Finally, the widely accepted exosomal markers CD9 (60232-1-Ig, Proteintech, China) and CD63 (25682-1-AP, Proteintech, China) protein expression [70] were detected.

### Cell-derived xenograft animal model

The 5-week-old Balb/c female nude mice were randomly divided into four groups. Each animal was intraperitoneally anesthetized with 150 mg/kg ketamine (Merial) and 6 mg/kg xylazine (Bayer). For the initial animal model, Hep-2 cells (200 µl, 1*10^7^/L) were injected and randomly divided into 3 groups (*n* = 6 per group), which received normal saline, cisplatin and M1-derived exosomes (100 µg per mice) every other day for 14 days. The HOTTIP-knockdown Hep-2 cells were injected as the negative control (*n* = 6). For the second model, Hep-2 cells that stably knocked down HOTTIP (*n* = 5) or the control vector (*n* = 15, 200 µl, 1*10^7^/L) were injected in aseptic conditions into the subcutaneous right axillary of the nude mice. Mice bearing the control vector cells were randomly divided into three groups, and normal saline (10 µl/g), cisplatin (2 µg/g) (P4394, Sigma, USA) and flagellin (1 ng/ml, 10 µl/g) (HSP-043, ProSpec, Israel) were administered at the peritumoral site every other day for 14 days. Finally, the mice were euthanized via cervical dislocation, after which the tumor volume (length*width^2^/2) and weight were calculated. All experimental animals were bred in a specific pathogen-free laboratory at 26–28 °C, with a 40–60% humidity and three mice per cage. Mice had free access to standard sterile feed and sterile drinking water. Artificial lighting was maintained at 10 h of light per day with 14 h of darkness.

### RNA isolation and real-time PCR analysis

First, 1 ml of TRIzol reagent (15596-026, Invitrogen, USA) was added to the samples, which were then ground in a TissueLyser II (Qiagen Company, Germany) for 10 min to obtain total RNA. Second, the total RNA was quantified using a spectrophotometer at an absorbance of 260 nm according to the manufacturer’s instructions. The 260/280 nm absorbance ratio ranged between 1.8 and 2.0. Next, 1 μg of total RNA was reverse-transcribed into cDNA, after which 2 μl of 1:10 diluted cDNA was amplified via PCR in a 20 μl reaction using SYBR Green Master Mix (CS7561, Invitrogen, USA). A CFX96 Real-Time PCR System (Bio-Rad) was used to perform quantitative real-time PCR. GAPDH was utilized as an internal control to normalize mRNA expression levels. PCR was performed on an Expand High Fidelity PCR System (Roche) under the following conditions: denaturation temperature of 95 °C for 45 s, annealing temperature of 60 °C for 45 s, and extension temperature of 72 °C for 1 min. The primer sequences are listed in Table 1.

**Table 1:** The primer sequences utilized in real-time PCR analysis.

	Forward	Reverse
Human HOTTIP	GGCTGGTGACATACTTCGCT	CACGGAGGGCAGGTGTATTT
Human TLR5	CCTTAGAGATGGCTGGTGCC	CCACCACCATGATGAGAGCA
Human MyD88	GCCGCCTGTCTCTGTTCTTGAA	GGTCCGCTTGTGTCTCCAGTTG
Human p65	CGCATCCAGACCAACAACAACC	AAGCAGAGCCGCACAGCATT
Human GAPDH	CAAATTCCATGGCACCGTCA	AGCATCGCCCCACTTGATTT

### Western blot analysis

The cells were lysed in equal volumes of ice-cold lysis buffer containing a protease inhibitor cocktail (Pierce Chemical Co.). Cell homogenates were boiled for 5 min in 5× Laemmli sample buffer. Total proteins were extracted with a Whole Protein Extraction kit (KGP250, KeyGEN BioTECH, China). Polyvinylidene difluoride membranes were then blocked in 5% fat-free milk or 5% BSA in TBS containing 0.05% Tween 20. Following overnight incubation at 4 °C with antibodies targeting TLR5 (ab13876, Abcam, UK), MyD88 (ab133739, Abcam, UK), p65 (ab32536, Abcam, UK), phosphorylated p65 (p-p65, ab86299, Abcam, UK) and GAPDH (ab181602, Abcam, UK), the membranes were incubated with HRP-conjugated secondary antibodies (KGAA35, KGAA37, KeyGEN BioTECH, China) at a 1:150,000 dilution for 1 h at room temperature and developed with a SuperSignal chemiluminescent detection system.

### Cell counting kit-8 (CCK8) assay

Cell Counting Kit-8 (CP736, DOJINDO Laborataries, Japan) was utilized. FaDu or Hep-2 cells were seeded in a 96-well plate (1000 cells per well). Next, 20 μl of CCK8 solution (5 mg/ml) was added to each well at the 0, 24, 48, 72 or 96 h time points. After an additional 2 h of incubation at 37 °C, the optical density (OD) value of each well was determined with a microplate reader (ELx800, BioTek, USA) at a wavelength of 450 nm.

### Cell proliferation assay (5-ethynyl-29-deoxyuridine, EdU)

The keyFluor555Click-iT EdU Kit (KGA337, KeyGEN BioTECH, China) was utilized. FaDu and Hep-2 cells were seeded in a 96-well plate (2 × 10^3^ per well) and incubated at room temperature for 30 min with 50 µL of cross-linking solution (PBS containing 4% paraformaldehyde). Each well was incubated with 50 µL of glycine (2 mg/ml) for 5 min with a decolorizing shaker, followed by 5 min of PBS washing. Next, each well was stained with 200 µl of 1×Apollo for 30 min at room temperature in the absence of light. The dye solution was washed 2–3 times by adding 100 ml of wash buffer (0.5% Triton X-100 in PBS) and shaking the plates for 10 min each time, after which the wash buffer was discarded. Reagent F was diluted with deionized water at a ratio of 100:1 to prepare a suitable amount of 1× Hoechst 33342 reaction solution. Each plate was incubated on a shaker at room temperature for 30 min, and the dye solution was discarded. Finally, each well was washed 1–3 times with 100 ml of PBS, after which 100 ml of PBS was added to preserve the cells. A high-content cell imaging system (MD, USA) was utilized for detection (magnification 200×).

### Flow cytometry

Cancer cells were suspended and collected, and the supernatant was discarded. According to the instructions of the Annexin-V-fluorescein isothiocyanate (FITC) cell apoptosis detection kit (400505, Biolegend, USA), Annexin-V-FITC, propidium iodide (PI), and binding buffer solution were prepared. Then, the cells (1 × 10^5^) were incubated for 15 min, and 500 µl of binding buffer was added. The fluorescence of FITC and PI was detected to analyze the apoptosis rate.

To evaluate macrophage polarization, the expression of CD14, CD86 and CD163 was assessed. The cells were dissociated at 4 °C with PBS-EDTA, resuspended in calcium- and magnesium-free PBS and incubated at 4 °C with an anti-CD14 primary antibody (human: 11-0149-42, eBioscience, USA; mice: 123307, Biolegend, American) for 1 h. After the cells were washed with PBS, they were incubated with a fluorescein-conjugated anti-mouse IgG secondary antibody at 4 °C for 45 min. Then, the cells were suspended in a PBS solution. The same procedures were utilized to stain for CD86 (human: 12-0869-42, eBioscience, USA; mice: 105007, Biolegend, American) or CD163 (human: 12-1639-42, eBioscience, USA; mice: 155305, Biolegend, American). Flow cytometry was performed on an LSRII (BD Biosciences) to detect the M1 (CD14^+^CD86^+^) and M2 (CD14^+^CD163^+^) phenotypes, and the data were analyzed using FlowJo Version X (TreeStar).

### Scratch test

FaDu and Hep-2 cells were seeded into 6-well plates at 5×10^5^ cells per well. The cells were scraped in the middle of each well after the cells completely adhered to the wall. Following incubation for 48 h, the migration distances and wound closure rates were calculated by biological inverted microscopy (OLYMPUS IX51, Japan).

### Transwell assay

To explore the invasion ability of cancer cells, the FaDu or Hep-2 cells were seeded into a 6-well plate. After the cells adhered to the wall, they were removed from the serum and cultured in an incomplete medium for 24 h. The Matrigel was placed at 4 °C overnight to allow melting and was then diluted twice with an incomplete medium. Next, 30 μL of the diluted Matrigel was added to the upper chamber of the Transwell and incubated at 37 °C for 120 min to polymerize the Matrigel into the glue. The cells were collected by digestion with 0.25% trypsin. One hundred microliters of the cell suspension (1 × 10^5^/ml) were placed into the upper Transwell chamber, and 500 µL of 20% FBS medium was added to the lower chamber. The 24-well cell culture plate was placed at 37 °C in a 5% CO2 incubator for 48 h. The Matrigel and cells in the upper chamber were removed, and the Transwell was then inverted and air dried. Next, 500 µL of 0.1% crystal violet was added to the chamber in the 24-well plate for 30 min at 37 °C. The cells were washed with PBS, and pictures were acquired (magnification 200×, OLYMPUS IX51, Japan) in three diameter fields.

We also established a co-culture Transwell system to study the influence of cancer cells on the polarization of THP-1-derived M0 macrophages. FaDu/Hep-2 cells (upper chamber) and M0 macrophages (lower chamber) co-cultures were performed for 72 h in DMEM medium in 6-well plates and 1 μm pore transwell inserts, preventing cell migration while allowing the diffusion of media components. The macrophages in the lower chamber of Transwell co-culture plates were collected for different experiments.

### Agilent miRNA chip

Total RNA containing small RNA was extracted from oe-HOTTIP FaDu-derived exosomes using TRIzol reagent (Invitrogen) and purified using the mirVana miRNA isolation kit (Ambion, Australia, TX, USA) according to the manufacturer’s protocol. Agilent miRNA array was used for miRNA analysis. Each slide of the Agilent array was designed as eight identical arrays (8 × 60 K format), and each array contained probes for detecting 2549 human mature miRNAs from miRBase R21.0. Each miRNA was repeatedly detected by the probe 30 times. The array also contained 2164 Agilent control probes. Microarray experiments were then performed according to the manufacturer’s instructions. Briefly, miRNA was labeled with Agilent miRNA labeling reagent. Total RNA (200 ng) was dephosphorylated and ligated with pCp-Cy3, and the labeled RNA was purified and hybridized with the miRNA array. The image was scanned with an Agilent microarray scanner (Agilent), gridded, and analyzed using Agilent feature extraction software version 10.10.

GeneSpring software V13 (Agilent) was used to analyze the miRNA array data for data aggregation, standardization and quality control. The default 90th percentile normalization method was performed for data preprocessing. Moreover, fold change thresholds of ≥2 and ≤ −2 were applied to select differentially expressed genes, and the Benjamini–Hochberg corrected p-value was 0.05. Next, the Adjust Data function of CLUSTER 3.0 software was used to log2 transform the data. The median value was then determined with the gene as the center, and hierarchical clustering was used with the average linkage degree for further analysis (Eisen et al., 1998). Finally, Java TreeView (Stanford University School of Medicine, Stanford, California, USA) was used to visualize the tree. The data was then uploaded to GEO (GSE163565).

### Dual-luciferase reporter gene assay

HEK 293T cells were seeded at 1 × 10^4^ cells/well in 96-well plates and cultured overnight. The next day, the cells were simultaneously transfected with pmiGLO-HOTTIP-WT (or -NC) or TLR5 3′-untranslated region (UTR)-WT (or -NC) reporter plasmids and hsa-miR-X mimic, inhibitor or their NC. After 48 h of transfection, the Dual-Luciferase Reporter Assay kit (E2920, Promega, USA) was utilized to detect the relative luciferase (RUC/Rluc) activity.

### Immunohistochemistry (IHC) and immunofluorescence (IF) assay

The cells or xenografts were fixed with 4% PFA, embedded in paraffin, and incubated at 4 °C overnight with primary antibodies against TLR5 (1:100, ab13876, Abcam, UK) and Ki67 (AB9260, MILLIPORE, USA). The samples were then washed, incubated with secondary antibody for 30 min at room temperature, and stained with diaminobenzidine. Positive reactions were defined as those showing brown signals in the cell cytoplasm. The intensity of staining was scored as follows: grade 0 = negative; grade 1 = weak; grade 2 = moderate; grade 3 = strong. The percentage ranking of positive cells: 0 = less than 5%; 1 = 5–25%; 2 = 26–50%; 3 = 51–75%; 4 = greater than 75%. A final score was calculated by multiplying the two scores. In terms of the IF assay, cells or xenografts were fixed with 4% PFA, washed three times with PBS, and treated with Triton X-100. The samples were then incubated overnight with CD11b (ab184308, Abcam, USA), iNOS (178945, Abcam, USA) or Arg-1 (96183, Abcam, USA) antibody (1:100) at 4 °C. The next day, they were washed with Tris-buffered saline and Tween 20 and stained with secondary antibodies (TRITC for CD11b, KGAA99, KeyGEN BioTECH, China and FITC for iNOS and Arg-1, Jackson ImmunoResearch 111-095-003, USA). A biological fluorescence microscope (Olympus BX43, Japan) was used to detect fluorescence. The image analysis software ImageJ (1.52o) was utilized for staining quantification by calculating the value of integral optical density (IOD).

### Enzyme-linked immunosorbent assay (ELISA)

The supernatant was obtained from stable cells after 48 h of incubation. ELISA was used to determine IL-1β, TNF-α, IL-6, IL-4 and IL-10 protein levels, and concentrations were calculated by comparison with standard curves. Briefly, 100 μl of buffer that was coated with monoclonal antibodies against IL-1β (KGEHC002b, KeyGEN BioTECH, China), TNF-α (KGEHC103α, KeyGEN BioTECH, China), IL-6 (KGEHC007, KeyGEN BioTECH, China), IL-4 (KGEHC006, KeyGEN BioTECH, China) and IL-10 (KGEHC009, KeyGEN BioTECH, China) was added to the wells of the plate. The plate was then covered with adhesive sealant and incubated for 2 h at room temperature on a microplate shaker. The solution was then aspirated, and the cells were washed with 300 μL buffer three times. Then, 100 μL of biotinylated detection antibody against IL-1β, TNF-α, IL-6, IL-4 or IL-10 was added. The second incubation was carried out at room temperature for 2 h in a plate microagglomerator. After adding 100 μl streptavidin conjugated with peroxidase, a third incubation was carried out at room temperature for 1 h in a plate microagitator. After washing, 100 μL of the tetramethylbenzidine substrate solution was added to each well. The plate was then incubated away from the light at room temperature for 5 min. After incubation, 100 μL of the stop solution (0.5 M HCl) was added to each well. The optical density of each well was determined using a microplate reader set to 450 nm.

### Statistical analysis

The number of biological replicates was presented by individual data points in each dot graph. The data were analyzed using SPSS software version 20.0 (SPSS, Inc., Chicago, IL, USA), for which the results were presented as the means ± standard deviations (SDs). ImageJ was used to conduct a quantitative analysis of IHC and IF staining. A two-tailed unpaired *t*-test and one-way analysis of variance (ANOVA) were conducted to compare the significance between or among groups. *P* values < 0.05 was considered to be statistically significant. Variance was similar between the groups that were being statistically compared.

## Supplementary information

Reproducibility Checklist

Supplementary Figure 1

Supplementary Figure 2

Supplementary Figure 3

Original Western blot of Figure 1E. CD9

Original Western blot of Figure 1E. CD63

Original Western blot of Figure 5C. TLR5, MyD88, p-p65

Original Western blot of Figure 5C. p65, GAPDH

Original Western blot of Figure 5G. TLR5, MyD88, GAPDH

Original Western blot of Figure 5G. p-p65, p65

Original Western blot of Figure 5I. TLR5, MyD88

Original Western blot of Figure 5I. p-p65, p65, GAPDH

## Data Availability

All data generated or analyzed during this study are included in this manuscript.
