# Supplementary figures and images for: M1 macrophage-derived exosomes and their key molecule lncRNA HOTTIP suppress head and neck squamous cell carcinoma progression by upregulating the TLR5/NF-κB pathway
Source: Cell Death Dis. 2022 Feb 24;13(2):183. doi: 10.1038/s41419-022-04640-z (PMC8873565; doi:10.1038/s41419-022-04640-z)

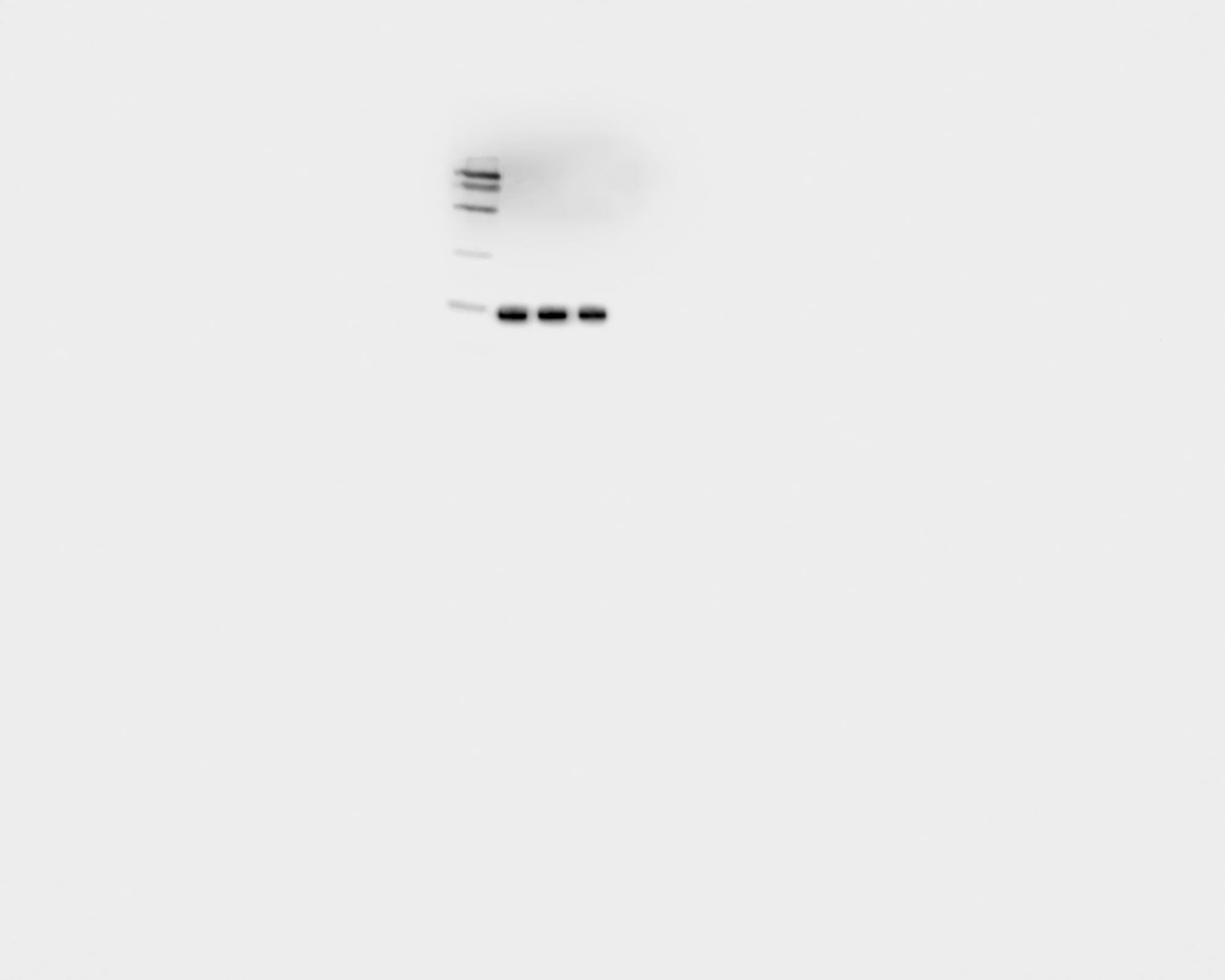

Supplement: Supplementary file 5 — Original Western blot of Figure 1E. CD9 [file 41419_2022_4640_MOESM5_ESM.tif]

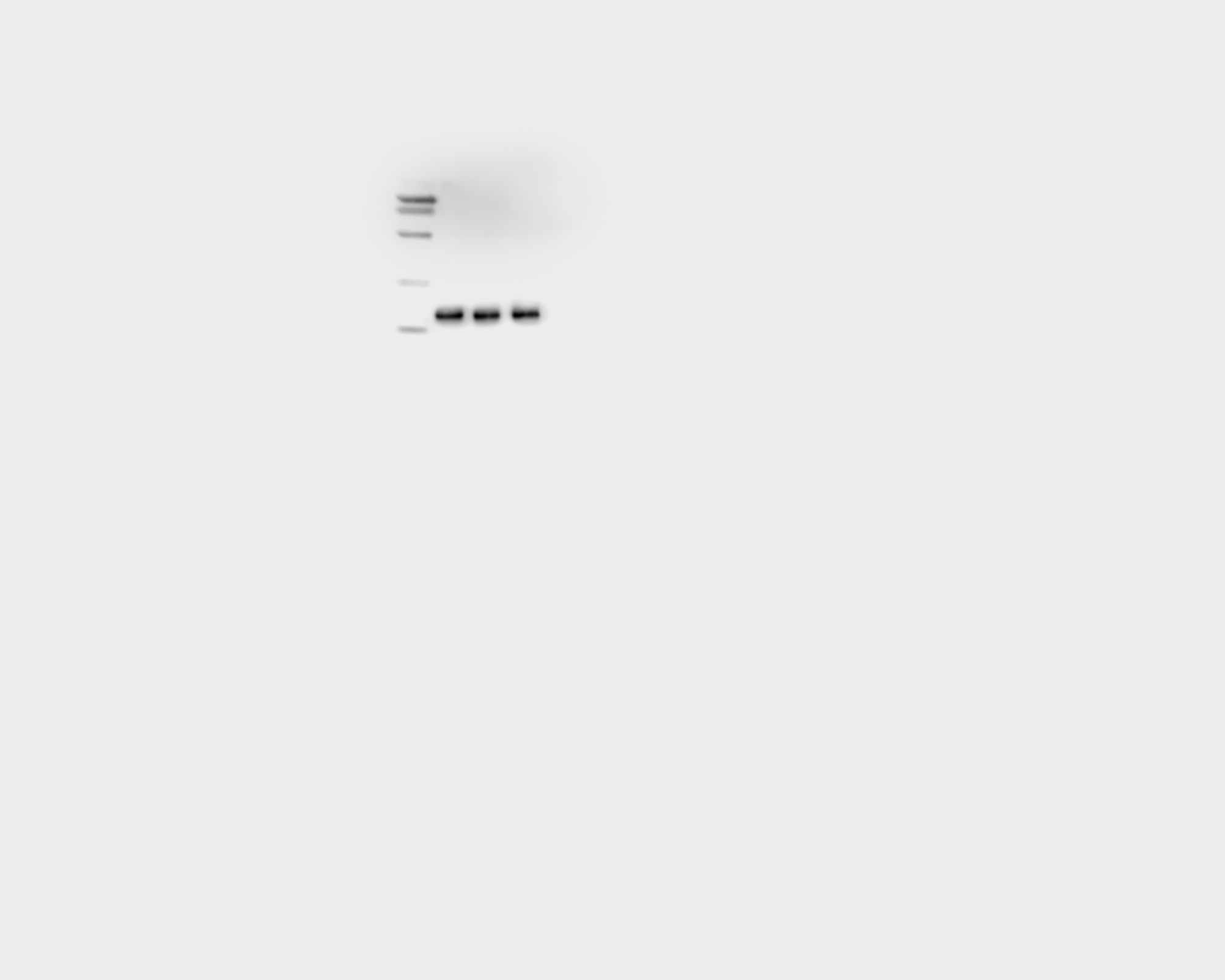

Supplement: Supplementary file 6 — Original Western blot of Figure 1E. CD63 [file 41419_2022_4640_MOESM6_ESM.tif]

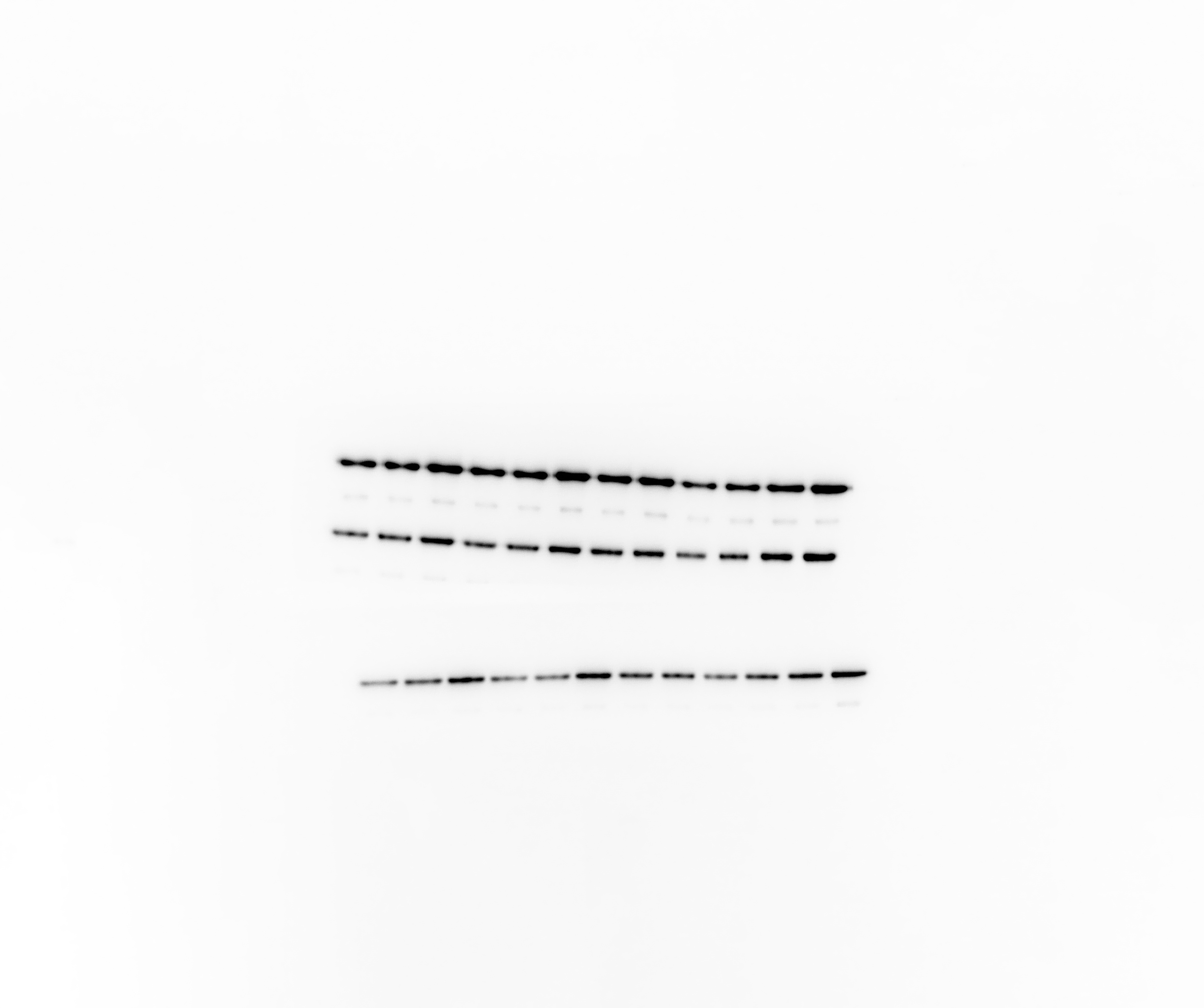

Supplement: Supplementary file 7 — Original Western blot of Figure 5C. TLR5, MyD88, p-p65 [file 41419_2022_4640_MOESM7_ESM.jpg]

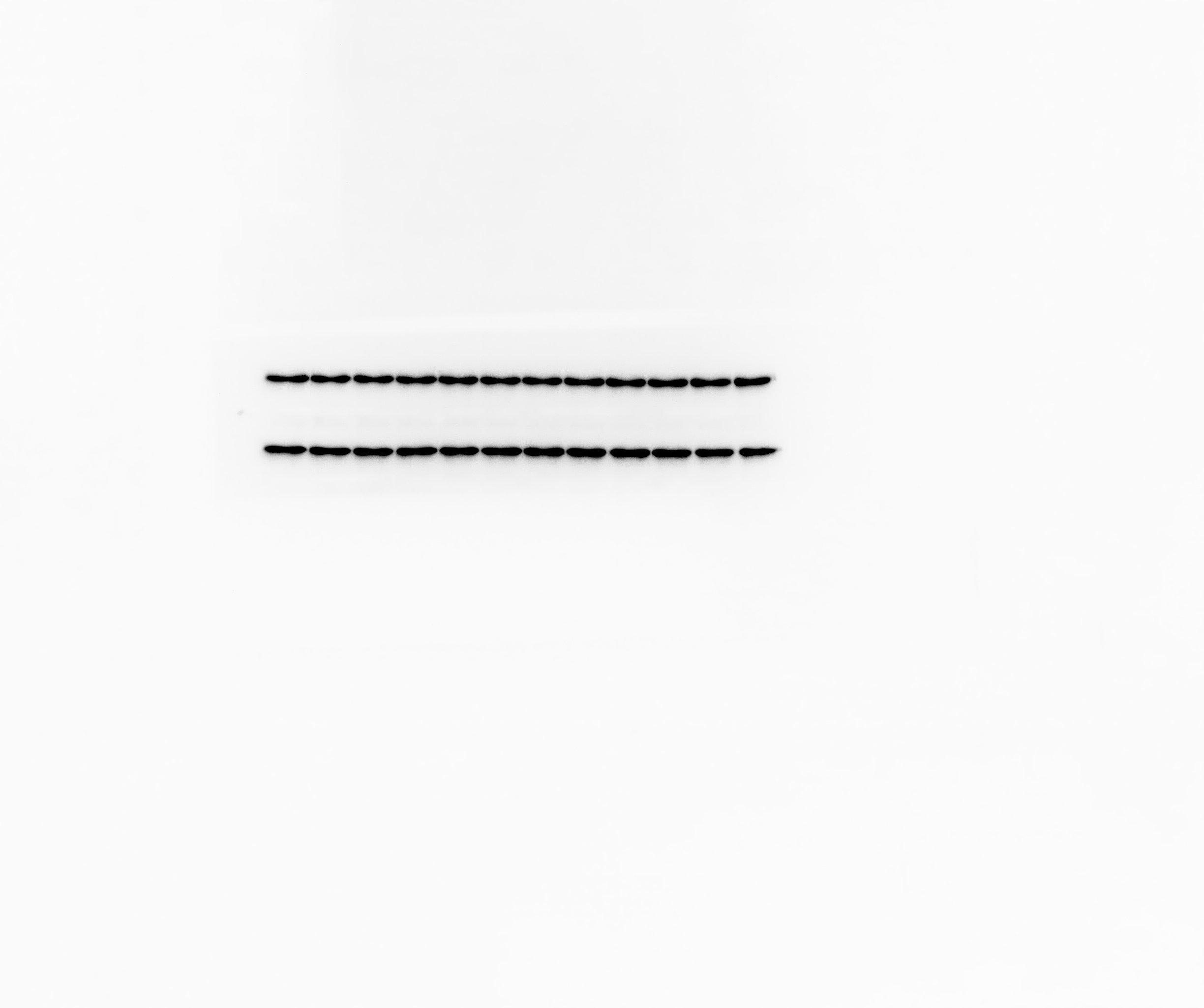

Supplement: Supplementary file 8 — Original Western blot of Figure 5C. p65, GAPDH [file 41419_2022_4640_MOESM8_ESM.jpg]

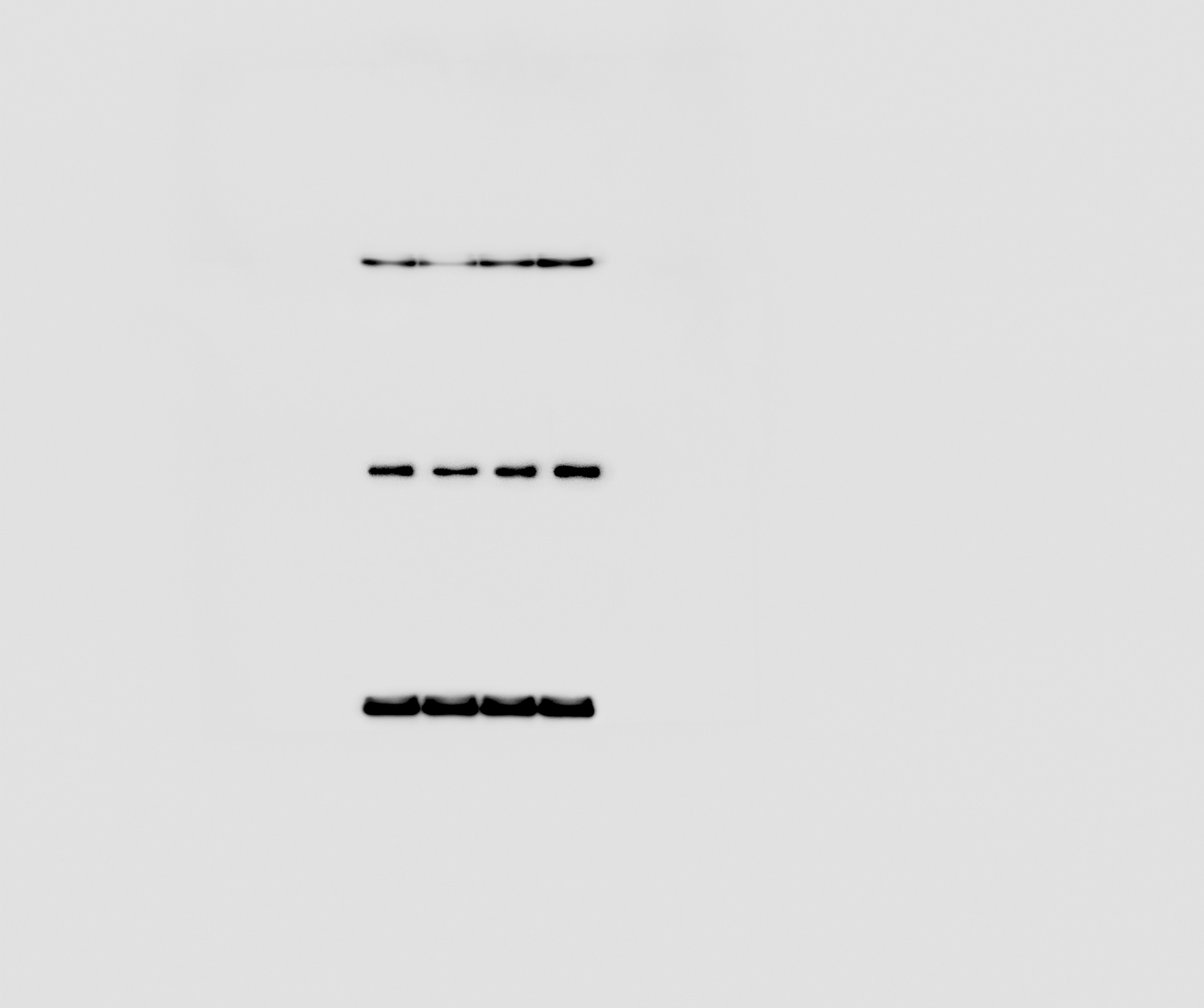

Supplement: Supplementary file 9 — Original Western blot of Figure 5G. TLR5, MyD88, GAPDH [file 41419_2022_4640_MOESM9_ESM.jpg]

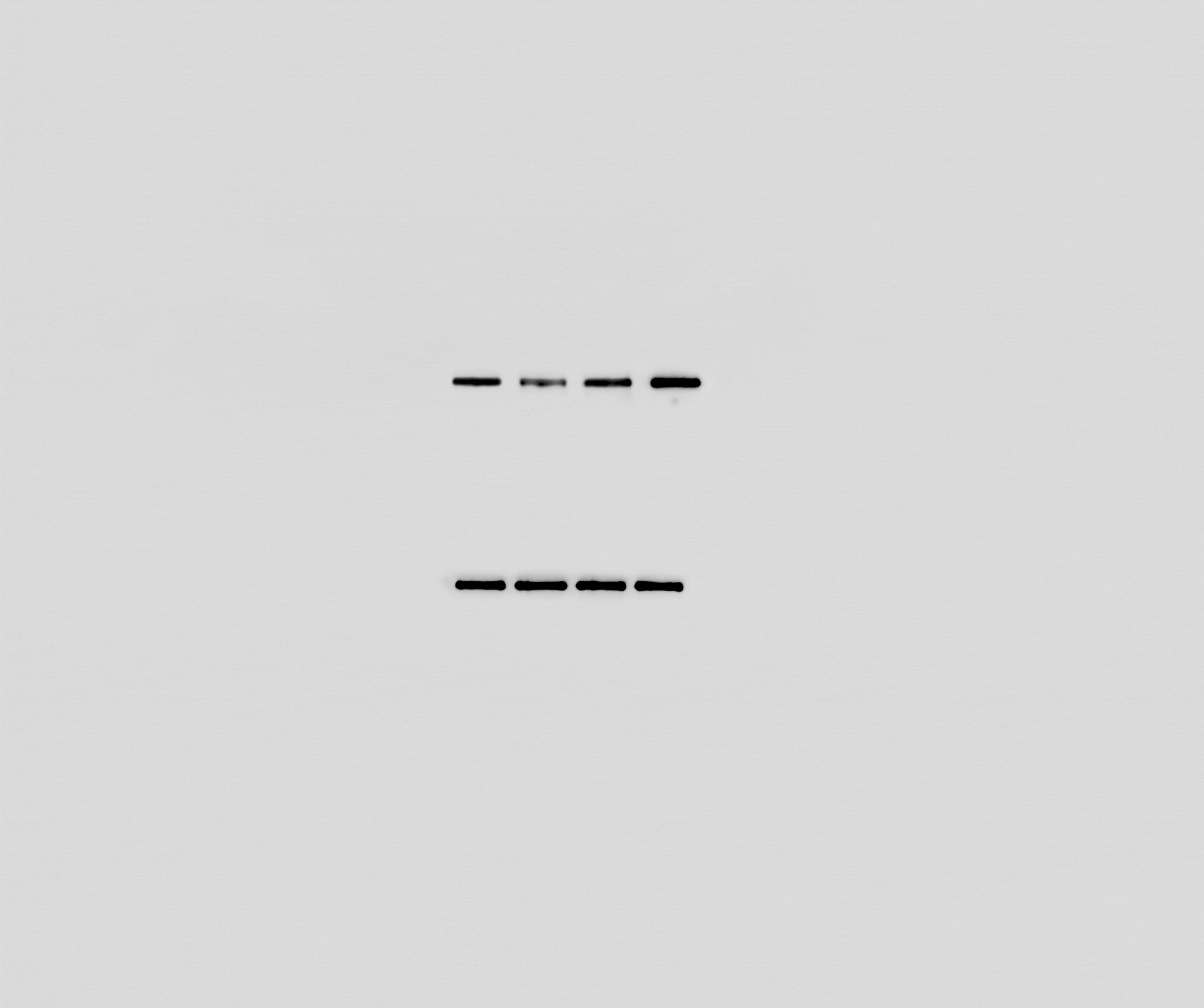

Supplement: Supplementary file 10 — Original Western blot of Figure 5G. p-p65, p65 [file 41419_2022_4640_MOESM10_ESM.jpg]

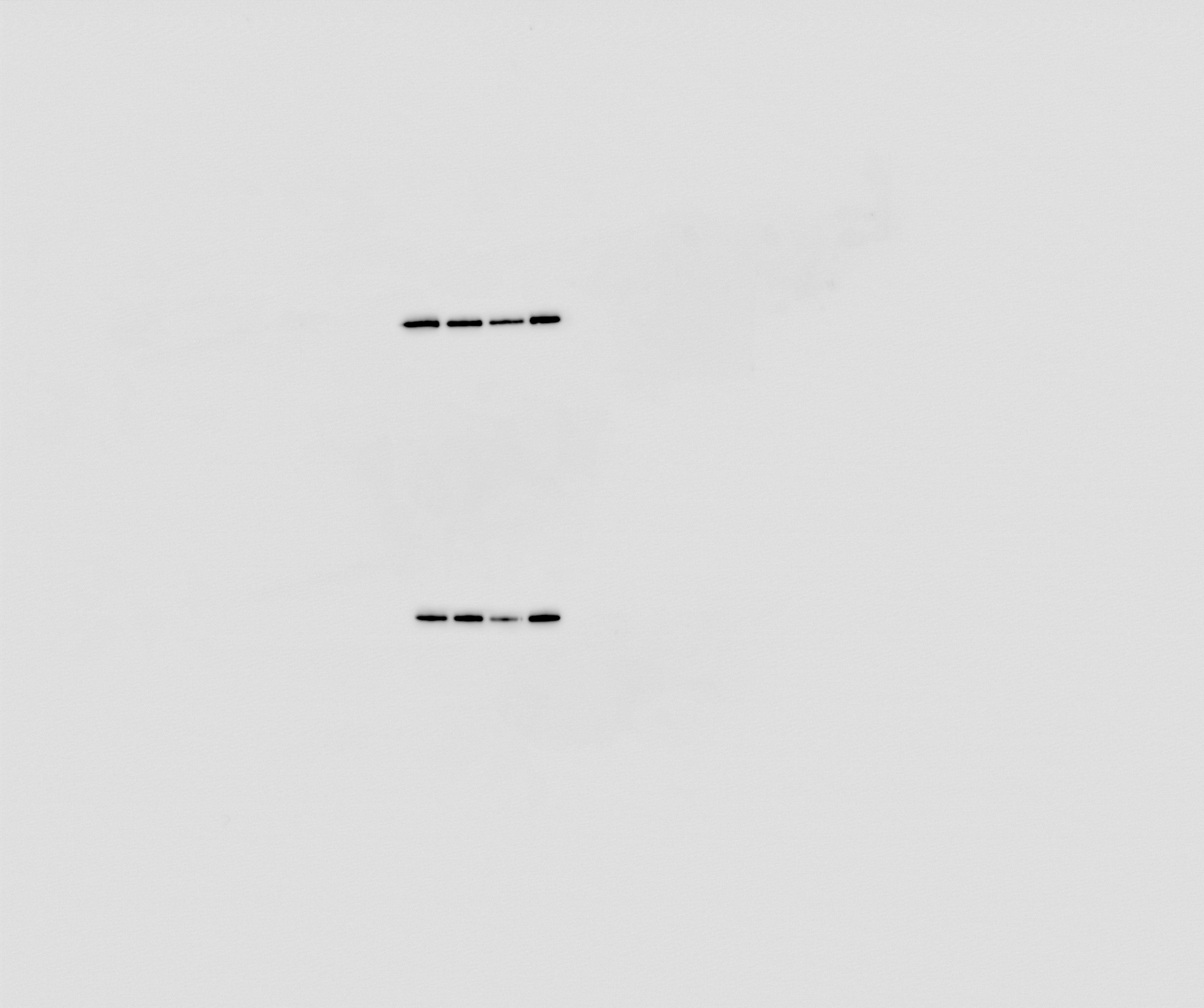

Supplement: Supplementary file 11 — Original Western blot of Figure 5I. TLR5, MyD88 [file 41419_2022_4640_MOESM11_ESM.jpg]

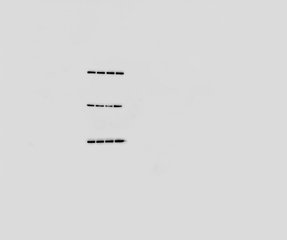

Supplement: Supplementary file 12 — Original Western blot of Figure 5I. p-p65, p65, GAPDH [file 41419_2022_4640_MOESM12_ESM.jpg]
